# The association and prognostic impact of enhancer of zeste homologue 2 expression and epithelial–mesenchymal transition in resected lung adenocarcinoma

**DOI:** 10.1371/journal.pone.0215103

**Published:** 2019-05-01

**Authors:** Taichi Matsubara, Gouji Toyokawa, Kazuki Takada, Fumihiko Kinoshita, Yuka Kozuma, Takaki Akamine, Mototsugu Shimokawa, Akira Haro, Atsushi Osoegawa, Tetsuzo Tagawa, Masaki Mori

**Affiliations:** 1 Department of Surgery and Science, Graduate School of Medical Sciences, Kyushu University, Fukuoka, Japan; 2 Department of Thoracic Surgery, Clinical Research Institute, National Hospital Organization Kyushu Medical Center, Fukuoka, Japan; 3 Clinical Research Institute, National Kyushu Cancer Center, Fukuoka, Japan; National Cancer Center, JAPAN

## Abstract

**Objectives:**

Epithelial-mesenchymal transition (EMT) and the histone methyltransferase Enhancer of Zeste Homologue 2 (EZH2) are important regulators of lung cancer progression and metastasis. Although recent studies support the correlation between EZH2 expression and EMT, no reports have investigated their association using immunohistochemistry or explored their prognostic impact on lung adenocarcinoma. The aim of this study was to elucidate the association between EZH2 and EMT, and their prognostic significance.

**Methods:**

EZH2 and the EMT markers E-cadherin and Vimentin were examined by IHC in lung adenocarcinoma specimens that were resected from 2003–2012. Associations between EZH2 and EMT markers and their correlations with survival were analyzed.

**Results:**

We enrolled 350 patients, approximately 70% of whom were diagnosed as pathological stage I. The rates of positive E-cadherin, Vimentin, and EZH2 expression were 60.3%, 21.4%, and 52.0%, respectively. There was a significant positive correlation between EZH2 and Vimentin expression (*p* = 0.008), and EZH2 scores were higher in the Mesenchymal group (*p* = 0.030). In multivariate analysis, EZH2 was an independent predictor of Vimentin expression, and *vice versa*. EMT and EZH2 overexpression were significantly correlated with poor disease-free and overall survival. Furthermore, the Epithelial group with high EZH2 expression had significantly worse disease-free and overall survival. Positive staining for EMT markers was unfavorable regarding disease-free survival among patients with low EZH2 expression.

**Conclusions:**

EMT and high EZH2 expression were associated with poor NSCLC prognoses. Vimentin is a key factor linking EMT and EZH2 in lung adenocarcinoma.

## Introduction

Lung cancer is the leading cause of cancer-related death worldwide. Non-small cell lung cancer (NSCLC) is the most common type of lung cancer, representing 85% of all cases, and is classified into several subtypes, including adenocarcinoma and squamous cell carcinoma[1]. The discovery of oncogenic driver mutations and their targeted therapies have become the forefront of NSCLC treatment[2, 3]. Furthermore, immune checkpoint inhibitors have emerged as a novel therapeutic strategy for NSCLC[4, 5]. Thus, therapeutic options for NSCLC have changed dramatically, and prognoses have improved compared with previous decades.

Epithelial-mesenchymal transition (EMT) changes a cell from having epithelial characteristics to mesenchymal characteristics. E-cadherin is an epithelial marker that maintains cell-cell adhesion and inhibits cell invasion[6]. Conversely, the mesenchymal marker Vimentin maintain intracellular mechanical homeostasis by mediating cytoskeleton architecture and the balance of cell force generation during EMT in cancer cells[7]. During EMT, E-cadherin expression is lost, while there is often a gain of Vimentin expression. EMT has been shown to play important roles in tumor invasion, metastatic spread and progression[8]. Furthermore, the genetic and epigenetic alterations that occur as cancer cells undergo EMT are currently being elucidated.

Regarding epigenetic dysregulation, histone methylation is one of the most important processes regulating the altered transcription associated with carcinogenesis[9]. The histone methyltransferase Enhancer of Zeste Homolog 2 (EZH2) catalyzes histone H3 lysine 27 (H3K27) trimethylation and represses transcription[10, 11]. Several reports have indicated that *EZH2* expression in NSCLC is associated with aggressive tumor phenotypes, advanced stage and poor survival [12]. Our previous report demonstrated that EZH2 positivity in lung adenocarcinoma was associated with higher metabolic activity in ^18^F-fluorodeoxyglucose positron-emission tomography/computed tomography (^18^F-FDG PET/CT)[13]. Thus, both *EZH2* expression and EMT contribute to tumor malignancy and metastatic activity. While several studies have investigated associations between *EZH2* expression and EMT, the clinical significance of *EZH2* expression and EMT in NSCLC has not been reported[14–16]. Thus, this study investigated correlations between EZH2 expression and the EMT status of resected lung adenocarcinoma specimens by immunohistochemical (IHC) staining, and their impacts on prognosis.

## Materials and methods

### Patients

We retrospectively examined 350 consecutive patients who underwent surgical resection for primary lung adenocarcinoma at the Department of Surgery and Science, Graduate School of Medical Sciences, Kyushu University between January 2003 and December 2012. Pathological stage was defined according to the criteria of the seventh edition of the International Association for the Study of Lung Cancer staging system. We investigated the following clinicopathological features: age at surgical resection, sex, smoking history, histological tumor grade, pathological tumor stage including lymph node metastases, pleural or lymphovascular invasion, and *epidermal growth factor receptor (EGFR)* mutation status (if available). After surgical resection, routine examinations, including blood tests (serum tumor markers) and chest radiography, were performed at 3-month intervals for the first 3 years and at 6-month intervals thereafter. CT scans were performed biannually for the first 3 years, and then at least annually thereafter. Written informed consent was obtained from each patient. This study was approved by Institutional Review Board at Kyushu University (No.: 28–380).

### IHC staining and evaluation

Formalin-fixed paraffin-embedded specimens were cut into 4-μm-thick sections, dewaxed with xylene, and rehydrated through a graded ethanol series. The IHC protocol for E-cadherin and EZH2 was as follows: (1) for antigen retrieval, sections were treated with Target Retrieval Solution (Dako, Glostrup, Denmark) at 115°C for 15 min after inhibiting endogenous peroxidase activity for 30 min with 3% hydrogen peroxidase in methanol; (2) sections were incubated with anti-E-cadherin monoclonal antibody (HECD-1, 1:1000; Takara, Shiga, Japan) or anti-EZH2 monoclonal antibody (clone 6A10, 1:100; Leica Biosystems, Newcastle, United Kingdom) at 4°C overnight; (3) immune complexes were detected with the Envision Detection System (Dako); and (4) sections were counterstained with hematoxylin. The Vimentin IHC protocol was as follows: (1) sections were incubated for 30 min in 3% hydrogen peroxidase in methanol without antigen retrieval; (2) sections were incubated with anti-Vimentin monoclonal antibody (clone V-9, 1:25; Dako) at room temperature for 60 min; (3) immune complexes were detected with the Envision Detection System (DAKO); and (4) hematoxylin was used as a counterstain.

E-cadherin expression was scored using the following previously reported criteria[17, 18]: (1) the proportion of stained tumor cells was scored as 0 (0%), +1 (1%–20%), +2 (21%–40%), +3 (41%–60%), or +4 (>61%); and (2) staining intensity was scored as +1 (weak), +2 (moderate), and +3 (strong). Both scores were then multiplied together to give a final E-cadherin staining score, among which, final scores ≥8 were considered positive for E-cadherin expression. For EZH2 IHC, tumor cells with nuclear staining were considered positive. All red scores were applied to discriminate between positive (score ≥3) and negative (score <3)[13, 19]. Vimentin IHC was scored according cytoplasmic staining in tumor cells; a positive score was defined as ≥3% of tumor cells, because nonspecific staining might be considered positive if positivity was defined as ≥1% of TCs. All evaluations were performed by at least two investigators. If independent assessments did not agree, the slides were reviewed by another investigator to obtain a consensus.

### Statistical analysis

Categorical variables are expressed as numerals, and continuous variables are expressed as means ± standard deviations. All statistical analyses were performed with JMP v13 (SAS Institute, Cary, NC, USA). For continuous variables, differences were evaluated using two-sided Student’s *t*-tests. For categorical variables, statistical differences between the expression of each molecule and patient characteristics were tested using χ^2^ or Fisher’s exact tests. Disease-free survival (DFS) was defined as the period between the date of initial surgery and the date of recurrence or death. Overall survival (OS) was defined as the period between the date of initial surgery and the date of the last follow-up or death. DFS and OS probabilities were estimated using the Kaplan–Meier method with the log-rank test. Univariate and multivariate analyses of relationships between EZH2 or Vimentin and clinicopathological characteristics were performed by logistic regression analysis with the backward elimination method. A *p*-value <0.05 was regarded as statistically significant.

## Results

### Patient characteristics and the expression of EMT markers and EZH2

Clinicopathological characteristics of the 350 lung adenocarcinoma patients are summarized in Table 1. There were 175 men and 175 women, with a median age of 69±9.3 years (range: 29–85); 178 (50.9%) were never smokers and 172 (49.1%) had a smoking history. The numbers of patients diagnosed as each pathological stage were as follows: IA 189 (54.0%), IB 65 (18.6%), IIA 28 (8.0%), IIB 25 (7.1%), IIIA 32 (9.2%), IIIB 7 (2.0%) and IV 4 (1.1%). *EGFR* mutation status was available for 216 patients, among whom, 113 (52.3%) were wild-type and 103 (47.7%) harbored *EGFR* mutations; most of the *EGFR* mutations were exon 19 deletions or L858R point mutations.

**Table 1:** Clinicopathological characteristics and expression of E-cadherin, Vimentin and EZH2 of the 350 enrolled patients.

Variables	No of patients (%)
Age (years)	69 ± 9.3
Sex	
Male	175 (50.0%)
Female	175 (50.0%)
Smoking history	
Never	178 (50.9%)
Smoker	172 (49.1%)
Histological grade	
G1	165 (47.2%)
G2/3	185 (52.8%)
pathological stage	
I	254 (72.6%)
II	53 (15.1%)
III	39 (11.2%)
IV	4 (1.1%)
pl	
Absent	275 (78.6%)
Present	75 (21.4%)
ly	
Absent	305 (87.1%)
Present	45 (12.9%)
v	
Absent	255 (72.9%)
Present	95 (27.1%)
*EGFR* status^a^	
Mutant	103 (47.7%)
Wild type	113 (52.3%)
E-cadherin	
Positive	211 (60.3%)
Negative	139 (39.7%)
Vimentin	
Positive	75 (21.4%)
Negative	275 (78.6%)
EMT phenotype	
Epithelial	174 (49.7%)
Intermediate	138 (39.4%)
Mesenchymal	38 (10.9%)
EZH2	
Positive	182 (52.0%)
Negative	168 (48.0%)

^a^cases in which data were available.

**Fig 1:**
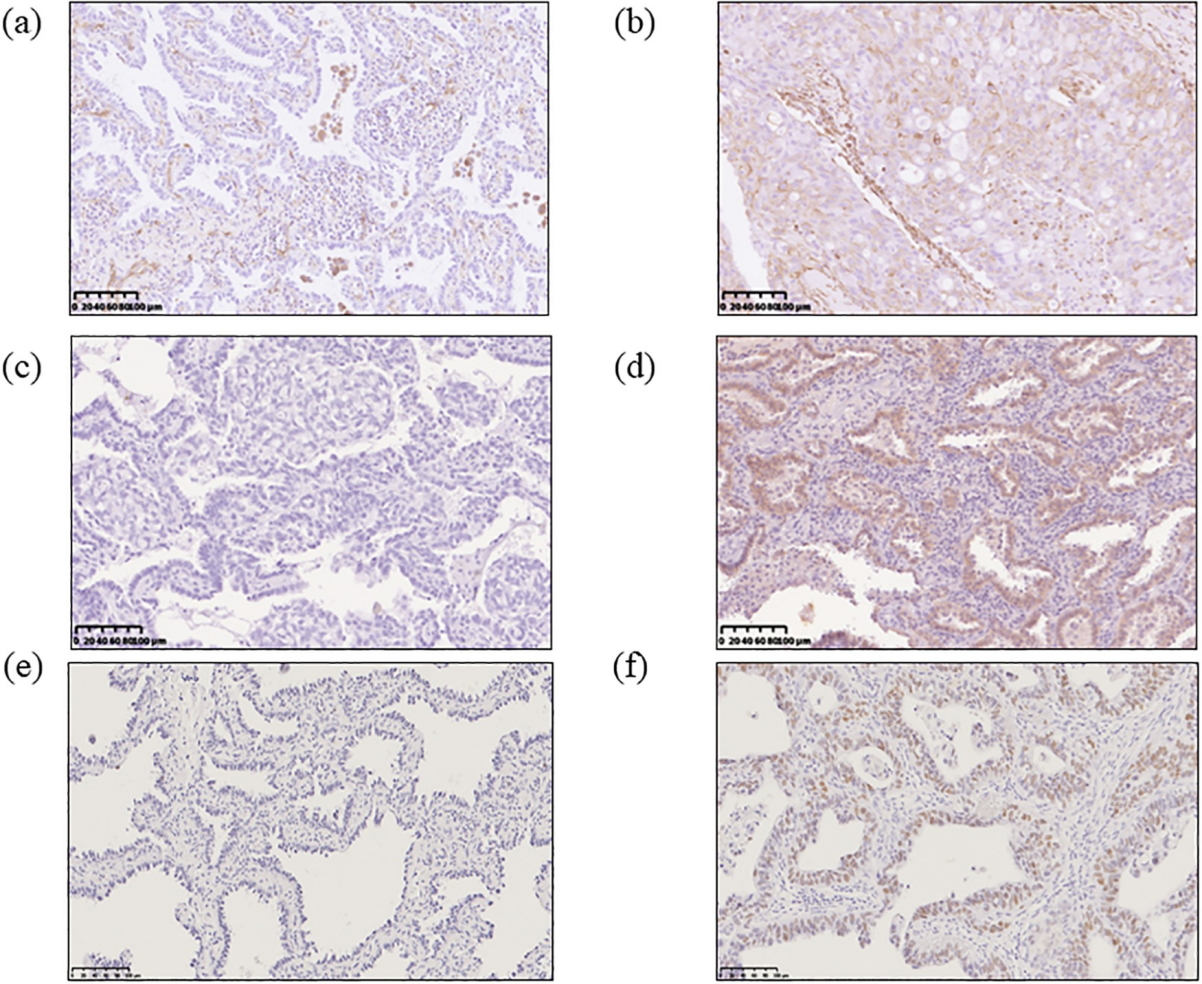
Vimentin, E-cadherin and EZH2 immunohistochemical (IHC) findings in lung adenocarcinoma specimens. IHC showing negative (a) and positive (b) Vimentin staining, which was detected in the cytoplasm of tumor cells. IHC showing reduced (c) and preserved (d) E-cadherin staining, which was primarily detected on the surface and in the cytoplasm of tumor cells. IHC showing negative (e) and positive (f) EZH2 staining, which was detected in the nuclei of tumor cells. Scale bar: 100 μm.

*EGFR*, *epidermal growth factor receptor*, EMT, epithelial-mesenchymal transition; EZH2, Enhancer of Zeste Homolog 2, G, histological grade; pl, pleural invasion; ly/v, lymphovascular invasion;

The frequencies of IHC positivity for E-cadherin, Vimentin and EZH2 are also shown in Table 1. The IHC analysis showed that 211 (60.3%) specimens were positive for E-cadherin (Fig 1a and 1b), 75 (21.4%) for Vimentin (Fig 1c and 1d), and 182 (52.0%) for EZH2 (Fig 1e and 1f). According to these results, we classified the 350 patients into three groups: Epithelial (E-cadherin positive and Vimentin negative), Intermediate (E-cadherin positive and Vimentin positive *or* E-cadherin negative and Vimentin negative), and Mesenchymal (E-cadherin negative and Vimentin positive). Thus, 174 (49.7%) patients were classified into the Epithelial group, 138 (39.4%) into the Intermediate group, and 38 (10.9%) into the Mesenchymal group.

### The association between EMT and EZH2

**Fig 2:**
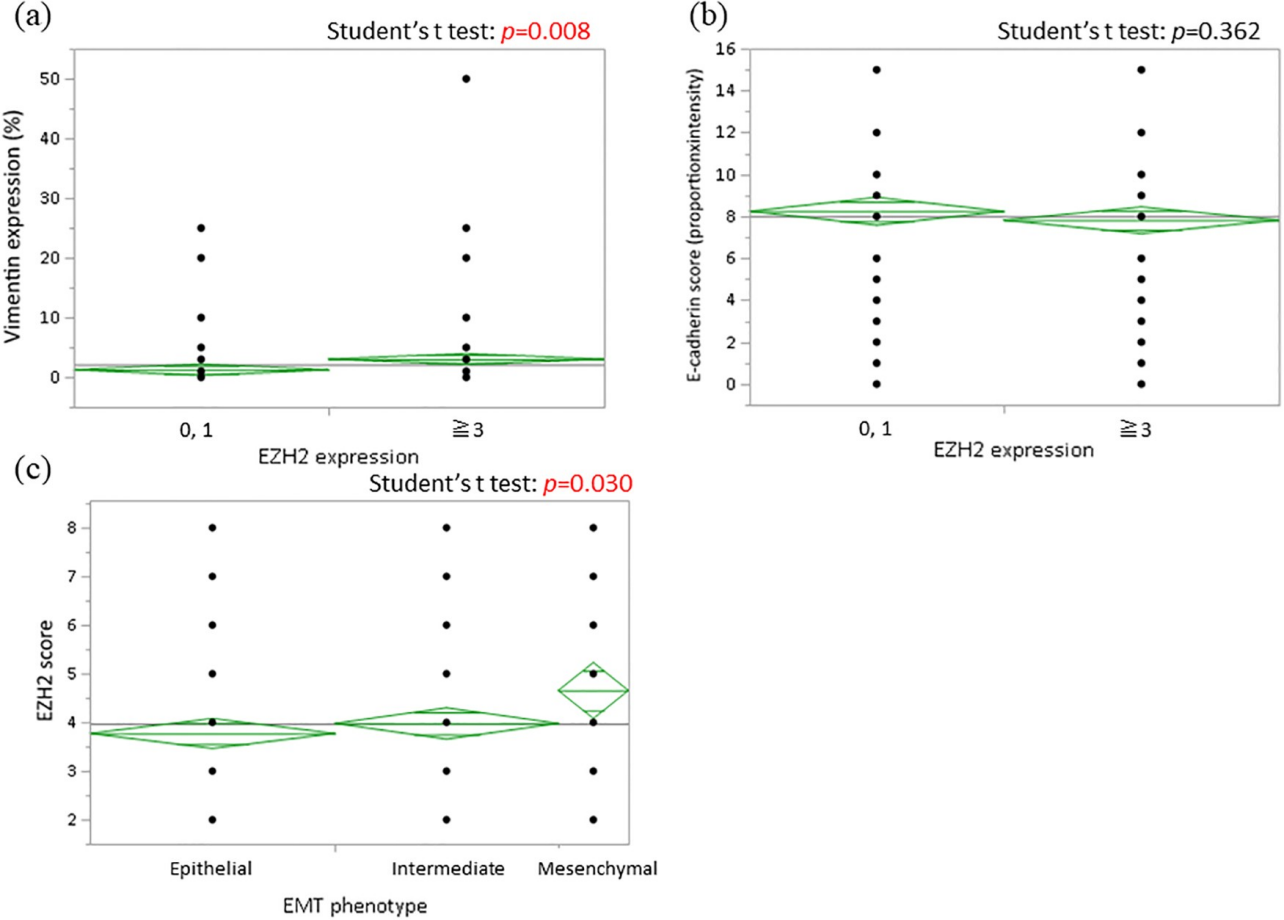
Analyses of associations between EZH2 expression and EMT markers. A significant positive correlation between EZH2 and Vimentin expression was identified (a), but there was no relationship between EZH2 and E-cadherin expression by student’s *t*-test (b). The mesenchymal group had a higher proportion of patients with positive EZH2 staining by student’s *t*-test (c).

We analyzed relationships between EMT markers and EZH2. Patients positive for EZH2 expression had a higher proportion of Vimentin positivity than those negative for EZH2 expression (*p* = 0.008), whereas there was no correlation between E-cadherin and EZH2 expression (*p* = 0.362) (Fig 2a and 2b). Next, we examined EZH2 expression within each EMT group. As shown in Fig 2c, the Mesenchymal group had significantly higher EZH2 expression than the other groups (*p* = 0.030). We further analyzed independent factors for EZH2 expression in patients with resected lung adenocarcinoma by univariate and multivariate analyses. In univariate analysis, positive EZH2 expression was associated with male sex, advanced stage, pleural and lymphovascular invasion, and Vimentin expression. In multivariate analysis, Vimentin expression was the only independent predictor of EZH2 expression. Additionally, multivariate analysis of Vimentin expression indicated that EZH2 and Vimentin were bidirectional independent predictors for each other’s expression (Table 2).

**Table 2:** Univariate and multivariate analyses of associations (A) between EZH2 expression and clinicopathological characteristics and (B) between Vimentin expression and clinicopathological characteristics.

(A)		Univariate analysis		Multivariate analysis		(B)		Univariate analysis		Multivariate analysis	
Factors		OR	*P* value	OR	*P* value	Factors		OR	*P* value	OR	*P* value
Age	<69	1.11	0.625			Age	<69	1.00			
(years)	≥69	1.00				(years)	≥69	1.30	0.315		
Sex	Female	1.00				Sex	Female	1.27	0.362		
	Male	1.58	0.033				Male	1.00			
Smoking	Never	1.00				Smoking	Never	1.00			
status	Smoker	2.06	<0.001			Status	Smoker	1.08	0.766		
Pathological	I	1.00				Pathological	I	1.00		1.00	
stage	≥II	3.41	<0.001			stage	≥II	2.64	<0.001	2.15	0.007
pl	Absent	1.00				pl	Absent	1.00			
	Present	2.52	<0.001				Present	2.49	0.002		
ly	Absent	1.00				ly	Absent	1.00			
	Present	3.76	<0.001				Present	2.59	0.0053		
v	Absent	1.00		1.00		v	Absent	1.00			
	Present	6.66	<0.001	6.18	<0.001		Present	2.32	0.002		
*EGFR*^a^	Wildtype	1.49	0.150			*EGFR*^a^	Wildtype	1.00			
	Mutant	1.00					Mutant	1.12	0.745		
Vimentin	Negative	1.00		1.00		E-cadherin	Negative	1.77	0.030		
	Positive	2.73	<0.001	2.28	0.006		Positive	1.00			
E-cadherin	Negative	1.25	0.302			EZH2	Negative	1.00		1.00	
	Positive	1.00					Positive	2.23	0.004	2.23	0.004

^a^cases in which data were available.

CI, confidence interval; *EGFR*, *epidermal growth factor receptor*; EMT, epithelial-mesenchymal transition; EZH2, Enhancer of Zeste Homolog 2; ly/v, lymphovascular invasion; OR, odds ratio; pl, pleural invasion

### Survival analyses with respect to EMT and EZH2 expression

**Fig 3:**
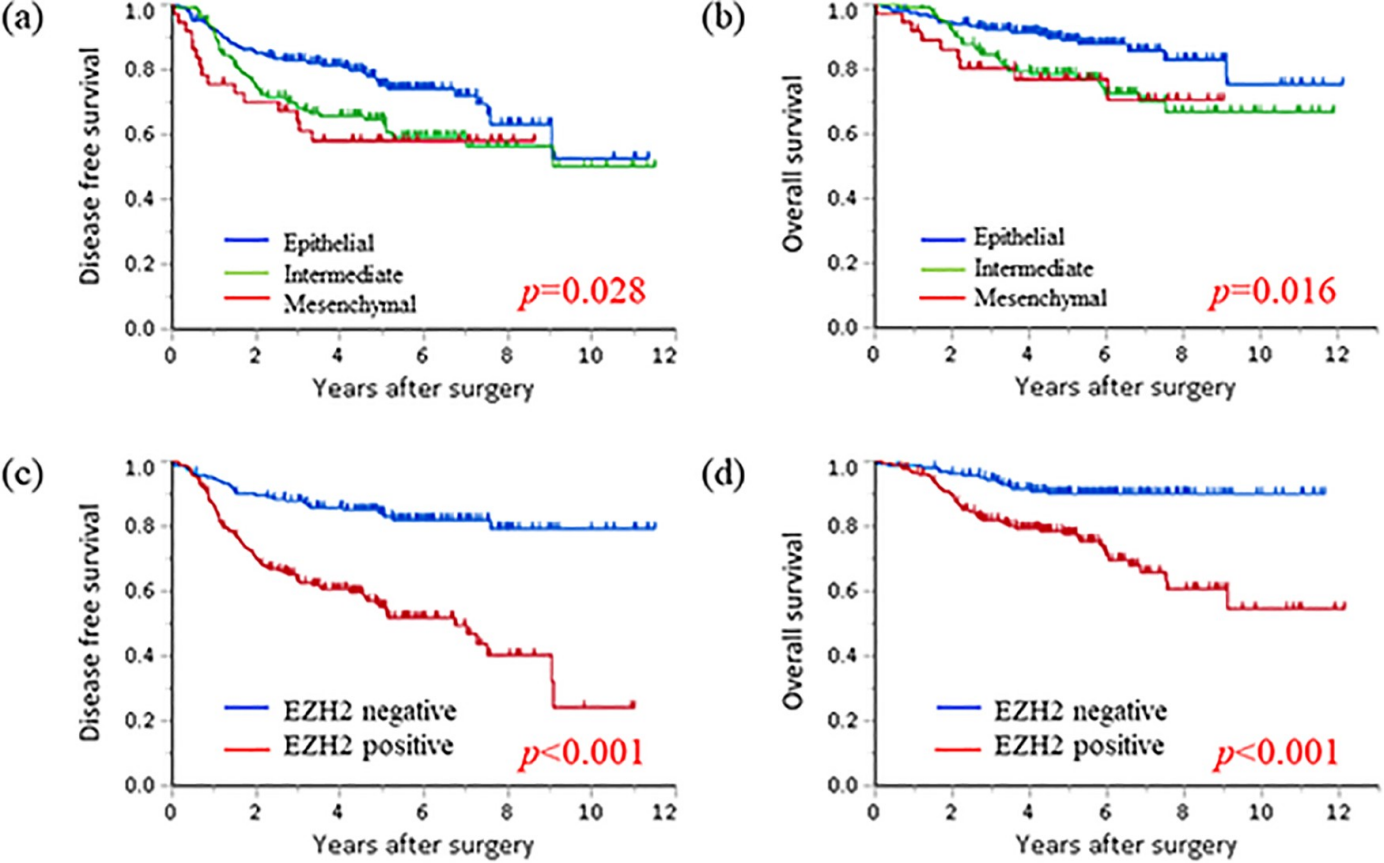
Kaplan–Meier survival curves according to EMT phenotypes and EZH2 expression. The Intermediate and Mesenchymal groups were associated with poor disease-free (a) (DFS: *p* = 0.028) and overall (b) (OS: *p* = 0.016) survival. Furthermore, positive EZH2 staining was also associated with poor disease-free (c) (*p*<0.001) and overall (d) survival (*p*<0.001).

**Fig 4:**
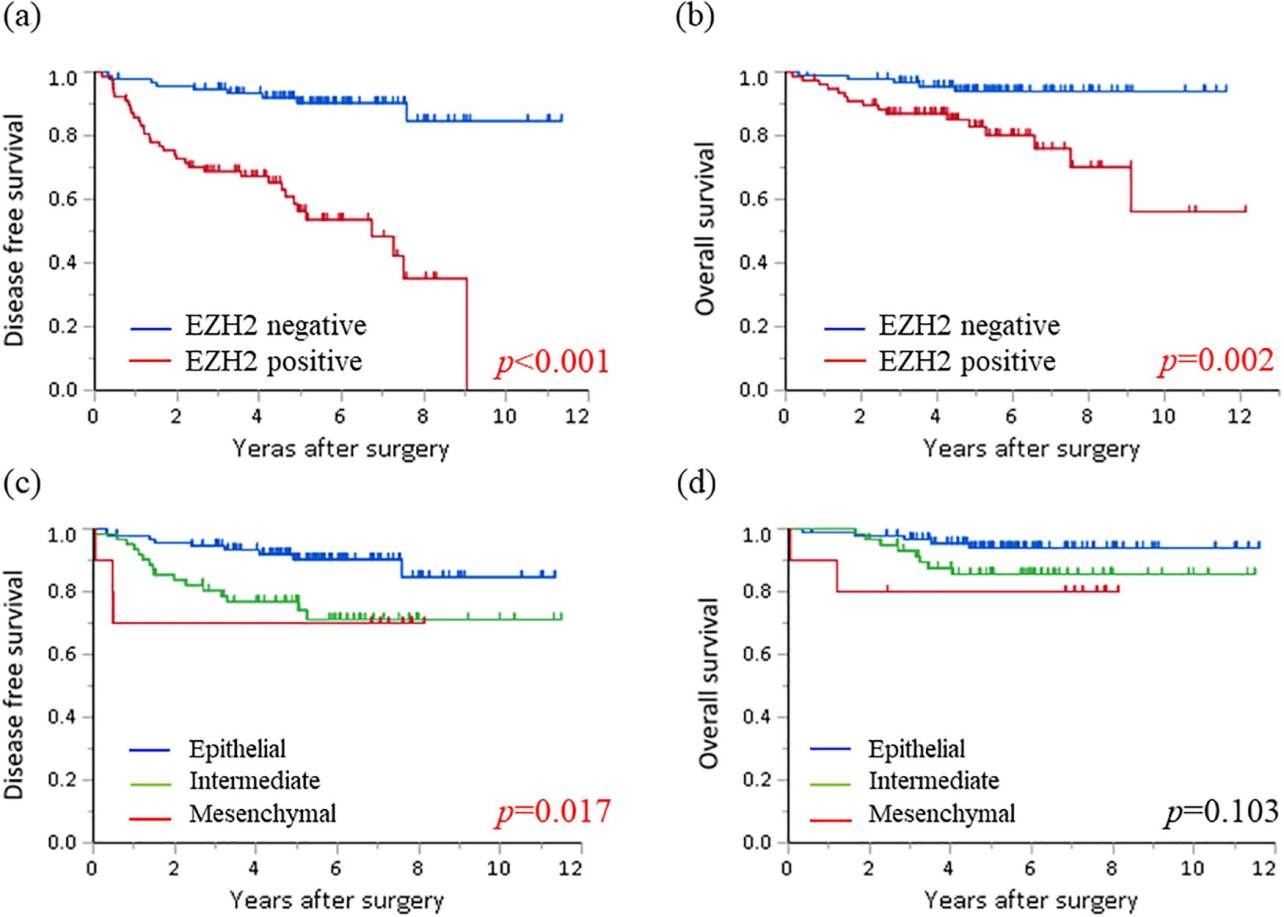
Kaplan–Meier survival curves of different EMT phenotypes in patients with negative EZH2 staining and those with positive EZH2 staining in the Epithelial group. In the Epithelial group, positive EZH2 staining was associated with significantly reduced disease-free (a) and overall (b) survival. In patients with negative EZH2 staining, EMT was significantly correlated with poor disease-free (c) but not with overall (d) survival.

The prognostic correlations of EMT phenotypes and EZH2 expression are shown in Fig 3. The Mesenchymal and Intermediate groups had poorer DFS and OS than the Epithelial group (log-rank test: *p* = 0.028 and *p* = 0.016, respectively). Patients with high EZH2 expression were significantly associated with poorer DFS and OS (log-rank test: *p*<0.001 for both). Furthermore, we investigated whether EMT and EZH2 were associated with survival rates for the groups with better prognoses. As shown in Fig 4, there were significant differences in the DFS and OS Kaplan–Meier curves among the Epithelial group when those patients were divided by EZH2 expression (log-rank test: *p*<0.001 and *p* = 0.002, respectively). Additionally, when patients were stratified by EZH2 expression, those with low EZH2 levels and the Intermediate or Mesenchymal phenotype were associated with unfavorable DFS. These results indicated that negative EZH2 expression and maintaining an epithelial phenotype were associated with the best prognoses. Furthermore, we analyzed the prognostic significance of EMT and EZH2 expression in patients divided into two subgroups, with Stage I or II-IV lung adenocarcinoma. In patients with Stage I disease, negative EZH2 expression and maintaining an epithelial phenotype were significantly associated with the best DFS and OS (S1 Fig). In patients with Stage II-IV disease, negative EZH2 expression and maintaining an epithelial phenotype were associated with better DFS but not OS. These results were similar to those shown in Fig 4, which included all the patients.

## Discussion

EMT is a crucial phenotypic change that facilitates tumor invasion and progression, and is associated with poor survival in NSCLC patients [17, 20]. EZH2 is an important factor associated with aggressive tumor behavior, advanced stage, and poor survival in NSCLC [12]. Several studies have investigated associations between EMT and EZH2; however, no studies have examined their correlation by IHC, nor have any determined their combined prognostic impact. Importantly, our study showed a significant correlation between EZH2 expression and EMT, and furthermore, demonstrated combined effects of EZH2 and EMT on survival in clinical lung adenocarcinoma specimens. In particular, the EMT marker Vimentin plays a key role linking EMT and EZH2.

Classic EMT characteristics include loss of epithelial polarity, loss of epithelial markers such as E-cadherin, and acquisition of mesenchymal markers such as Vimentin[21]. According to IHC results, we classified the 350 patients into three groups: Epithelial (E-cadherin positive and Vimentin negative), Intermediate (either loss of E-cadherin alone or gain expression of Vimentin alone), and Mesenchymal (E-cadherin negative and Vimentin positive). Recent studies have suggested that EMT involves not only genetic factors but also epigenetic remodeling, including altered DNA methylation and histone modification[22, 23]. The methyltransferase EZH2 is the catalytic subunit of Polycomb Repressive Complex 2 (PRC2), which methylates H3K27, repressing transcription[10, 11]. Our results revealed that EZH2 expression was higher in the Mesenchymal group than in the other two groups. In univariate and multivariate logistic regression analyses, Vimentin and EZH2 were mutually independent factors associated with the expression of the other.

Whether there are common underlying mechanisms for EMT and EZH2 expression remains unclear. Several studies have demonstrated associations between EZH2 and EMT *in vitro* and *in vivo*. EZH2 repress transcription of several molecules including E-cadherin [24]. In this process, some long non-coding RNAs (lncRNAs) have been shown to potentially link EMT and EZH2 [16, 25, 26]. Battistelli *et al*. found that Snail, which is an EMT repressor, regulated EZH2 activity in hepatocytes *via* the lncRNA HOTAIR. Additionally, the Snail/HOTAIR/EZH2 complex was found to regulate Snail activity *in vivo*. Taking the results of Wen *et al*. and Sun *et al*. together, it appears that EZH2 promotes EMT in NSCLC cells by inhibiting the lncRNA SPRY-IT1, inducing invasion, metastasis, and proliferation. In their studies, siRNA-mediated knockdown of lncRNA SPRY-IT1 increased EZH2 mRNA and protein expression, and promoted cell invasion and migration. Furthermore, E-cadherin expression was notably decreased and Vimentin was increased in the si-SPRY-IT1 group. In contrast, EZH2 knockdown increased E-cadherin and decreased Vimentin expression. In the present study, we demonstrated that higher EZH2 expression was associated with EMT, especially vimentin expression. Although this study did not reveal the detailed molecular mechanisms behind this association, we believe that our findings by IHC using patient specimens support these previous reports [16, 25, 26] and suggest future therapeutic strategies.

The Intermediate and Mesenchymal groups had unfavorable prognoses compared with the Epithelial group in our survival analyses (DFS: *p* = 0.028, OS: *p* = 0.016). Several reports have analyzed NSCLC survival according IHC for E-cadherin and Vimentin. For example, Sowa *et al*. investigated E-cadherin and Vimentin expression and their correlations with lung adenocarcinoma prognosis[27]. Like this study, they classified 239 patients by E-cadherin and Vimentin expression, and investigated their prognosis. They found that the “complete EMT conversion group” (corresponding to our “Mesenchymal group”) had the worst prognosis and the “no EMT conversion group” (corresponding to our “Epithelial group”) had the best prognosis. Tsoukalas *et al*. examined the prognosis of NSCLC patients along with the expression of E-cadherin and Vimentin[28]. In their study, there was a positive correlation between E-cadherin expression and survival and a negative correlation between Vimentin expression and survival. These and our results indicated that both negative E-cadherin and positive Vimentin staining are prognostic factors, and that these factors affect prognosis independent of fully completing EMT (E-cadherin negative and/or Vimentin positive).

Finally, we investigated whether EZH2 expression and EMT mutually affected the prognosis of lung adenocarcinomas. As shown in Fig 3, negative EZH2 expression and the Epithelial group had favorable prognoses. However, within the Epithelial group, EZH2 was still an important factor associated with poor prognosis (DFS: *p*<0.001, OS: *p* = 0.002). Additionally, IHC results consistent with EMT indicated worse DFS (*p* = 0.0173). EZH2 was recently confirmed to be a therapeutic target, as the EZH2 inhibitor Tazemetrostat showed antitumor activity against refractory B-cell lymphoma and advanced solid tumors in phase I clinical trials [29]. Takashina *et al*. showed that combined inhibition of EZH2 and histone deacetylases had a synergistic antiproliferative effects on NSCLC cells, and that cotreatment suppressed *in vivo* tumor growth [30]. As we identified a correlation between EZH2 and EMT, especially regarding Vimentin expression, targeting EZH2 is a possible therapeutic approach for blocking EMT.

There were some limitations to this study. First, this was a retrospective study with a larger proportion of stage I patients (74.6%) than is generally reported[31]. Patients with advanced disease had a significantly higher rate of mesenchymal phenotypes than stage I patients in our study, so it is possible that the disparity of this population effected our results. Second, we classified EMT status using IHC for two EMT markers, E-cadherin and Vimentin. Several additional transcription factors can induce EMT, including SNAIL1/2, ZEB1/2 and TWIST; thus, it may be possible to analyze associations and underlying mechanisms between EZH2 and EMT more accurately by evaluating other EMT markers. Next, there are several epigenetic regulators other than EZH2, which are reported to be associated with poor survival and to be regulator of EMT in lung cancer [32, 33]. Furthermore, several downstream molecules of EZH2 expression are reported to predict lung cancer prognosis [34]. It is possible that EZH2 is also associated with poor survival through some mechanism other than EMT. Further studies are needed to investigate the clinicopathological and prognostic significance of other epigenetic regulators or downstream genes of EZH2 expression.

In conclusion, using IHC, we demonstrated a significant correlation between EZH2 and EMT and their unfavorable correlations with lung adenocarcinoma prognosis. Vimentin was an important factor linking EMT with EZH2 expression. Further investigation of these two prognostic factors will help develop future lung cancer treatments.

## Supporting information

S1 Fig Kaplan-Meier survival curves according to EMT phenotype and EZH2 expression. In the subgroup of patients with Stage I lung adenocarcinoma, negative EZH2 expression without EMT conversion was significantly associated with the best disease-free (a) and overall (b) survival. In the subgroup of patients with Stage II-IV disease, negative EZH2 expression and maintaining an epithelial phenotype were associated with better disease-free (c), but not overall (d) survival.
(TIF)
