# Supplementary figures and images for: The association and prognostic impact of enhancer of zeste homologue 2 expression and epithelial–mesenchymal transition in resected lung adenocarcinoma
Source: PLoS One. 2019 May 1;14(5):e0215103. doi: 10.1371/journal.pone.0215103 (PMC6493717; doi:10.1371/journal.pone.0215103)

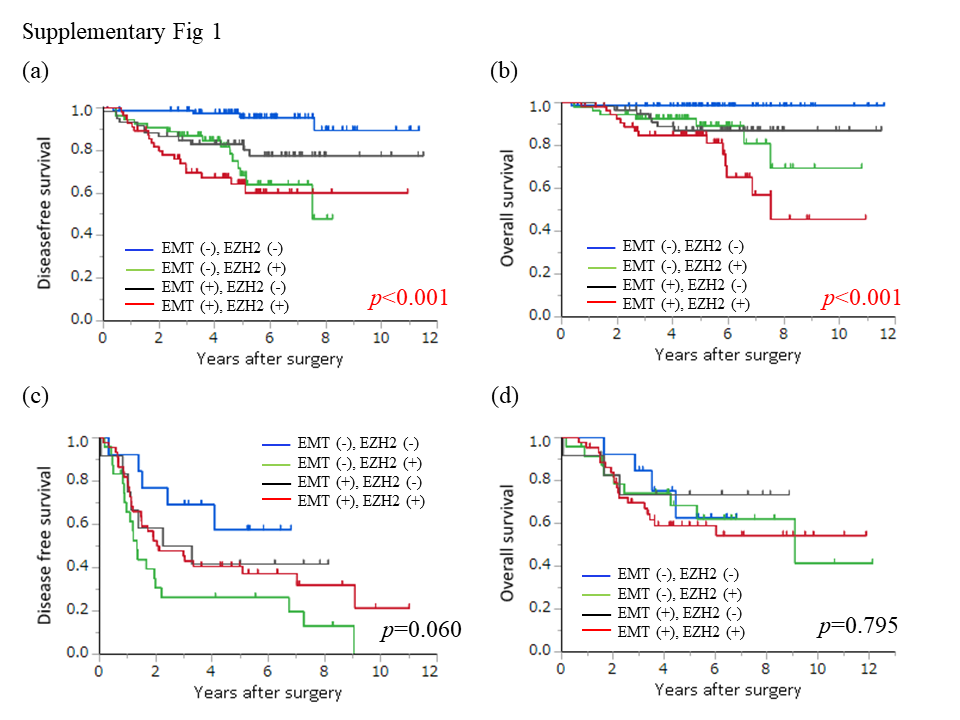

Supplement: S1 Fig — In the subgroup of patients with Stage I lung adenocarcinoma, negative EZH2 expression without EMT conversion was significantly associated with the best disease-free (a) and overall (b) survival. In the subgroup of patients with Stage II-IV disease, negative EZH2 expression and maintaining an epithelial phenotype were associated with better disease-free (c), but not overall (d) survival. (TIF) [file pone.0215103.s001.tif]
